# The Effectiveness of a Mobile Health Care App and Human Coaching Program in Primary Care Clinics: Pilot Multicenter Real-World Study

**DOI:** 10.2196/34531

**Published:** 2022-05-06

**Authors:** HyoRim Ju, EunKyo Kang, YoungIn Kim, HyunYoung Ko, Belong Cho

**Affiliations:** 1 Department of Family Medicine Seoul National University Hospital Seoul Republic of Korea; 2 National Cancer Control Institute National Cancer Center Goyang Republic of Korea; 3 Noom Korea Noom Inc. Seoul Republic of Korea

**Keywords:** healthcare, health care, mobile application, app, self-management, primary care, chronic conditions

## Abstract

**Background:**

As the global burden of chronic conditions increases, their effective management is a concern. Although the need for chronic disease management using mobile self-management health care apps is increasing, there are still many barriers to their practical application in the primary care field.

**Objective:**

This study evaluated the effectiveness of primary care services combining a mobile self-management health care app with human coaching for patients with chronic diseases in the current primary care system.

**Methods:**

A total of 110 patients (mean age 53.2, SD 9.2 years; 64 of 110, 58.2% female) with hypertension, diabetes, dyslipidemia, or metabolic syndrome who visited one of 17 participating primary care clinics from September to November 2020 were included in this study. All participants recorded data regarding changes in body weight, sleep conditions, quality of life, depression, anxiety, stress, BMI, waist circumference, blood sugar levels, blood pressure, and blood lipids levels. The app user group (n=65) used a mobile self-management health care app with human coaching for 12 weeks, and the control group (n=45) underwent conventional self-managed health care.

**Results:**

Patients in the app user group reported significantly more weight loss than those in the control group—the body weight of the app user group decreased by 1.43 kg (95% CI –2.07 to –0.79) and that of the control group decreased by 0.13 kg (95% CI –0.67 to 0.41; *P*=.002). The weight loss was markedly greater after using the app for 9 weeks than that when used for 4 weeks or 5-8 weeks (*P*=.002). Patients in the app user group reported better sleep quality (*P*=.04) and duration (*P*=.004) than those in the control group.

**Conclusions:**

The combination of primary care clinics and a mobile self-management health care app with human coaching results in better management of chronic conditions. This study shows that the primary care services combining a mobile self-management health care app with human coaching are effective in the current primary care system. An implication of this study is the possibility that a mobile self-management health care app with human coaching is a treatment option in the current primary care system.

## Introduction

The burden of chronic conditions has increased markedly [1], and the incidence of conditions such as hypertension, diabetes, and hyperlipidemia is rising in South Korea. Health care systems worldwide are challenged with clinical and economic burdens of chronic and complex conditions, resulting in major obstacles in the provision of optimal health care [2]. Determining the potential benefits of lifestyle modifications and patient participation in health-related decisions is necessary owing to the increased demands on primary care clinics and health care systems [3,4].

The effects of lifestyle modifications on patients with chronic conditions have been identified in previous studies [5-9]. However, a model that supports lifestyle modifications in an evidence-based manner in a format that can be integrated into clinical practice is necessary for primary care providers. Patient involvement is critical for the clinical integration of such models; patient participation in treatment planning, knowledge exchanges, setting goals, and performing self-care activities is necessary for effective lifestyle modifications [10,11]. Patient participation is valuable for symptom control and the management of chronic health conditions [12]. Self-management strategies for lifestyle modifications are increasingly recognized as important tools for chronic disease management and secondary prevention [13].

Technical innovations have increased access and improved health care quality as they have enabled the dissemination and improvement of health care via nontraditional channels at an unprecedented rate with the removal of practical barriers [14]. Technical innovations, such as the transmission of medical records, teleconsulting, telemonitoring, telemedicine, and teleprescription, have led to the development and utilization of tools to promote lifestyle modifications in the health care industry [15,16]. Strategies for implementing lifestyle modifications include self-management aimed at behavior changes, educational interventions, and motivation to participate in self-management [17]. Mobile health technology allows mobile devices, such as tablets, smartphones, and laptops, to play an important role in the collection, storage, and transmission of health data; supports real-time monitoring and the self-management of patients; and has made a huge difference in lifestyle modification interventions [18,19]. Previous studies have shown the efficacy of lifestyle modifications using various mobile tools [15,20,21]. A meta-analysis that evaluated the effectiveness of mobile self-management health care apps for lifestyle modifications in patients with type 2 diabetes found that the use of most apps resulted in significant changes in hemoglobin A_1c_ (HbA_1c_) levels [15]. Another study reported that combining a health care app with a wearable device result in lifestyle modifications that affect the BMI and cholesterol level of patients [20]. Moreover, digital interventions using technologies, such as the internet, SMS, software applications, and mobile sensors may improve positive behavioral factors (physical activity, diet, and medication adherence), and these are even more effective when used to treat multiple behavioral outcomes simultaneously [21].

The lifestyle modification tools provided with recent technological advances allow for sustainable changes by supporting self-care and providing more personalized health care. However, previous studies regarding these tools are limited. Primary health care physicians are usually the first point of contact between the health care system and patient [22], including patients with chronic diseases who utilize the health care system regularly. However, evidence-based models, especially those using information technology, are currently not available for primary care providers to effectively support lifestyle modifications and promote changes in patient behavior. Most previous studies include patients from clinical settings who are already using health care tools or those who are exceptionally motivated to do so [23,24]. Previous studies suggested an integrated whole-systems approach at the patient, primary care, and service organization levels [25]. The effective implementation of these models is necessary for ensuring feasibility, sustainability, and scalability [26]. However, not all patients are eligible for participation in these models owing to their socioeconomic status, lack of access to the internet, and other technologies. Moreover, systematic and comprehensive data on the implementation and utilization aspects of mobile self-management apps, especially in primary care settings, are lacking.

This study evaluated the effectiveness of primary care services combining a mobile self-management health care app with human coaching in the current primary care system for patients with hypertension, diabetes, dyslipidemia, or metabolic syndrome, and on changes in body weight, sleep condition and quality of life, mental health (depression, anxiety, and stress), and cardiovascular risk factors (BMI, waist circumference, blood sugar level, blood pressure, and blood lipid levels).

## Methods

### Study Population

Patients aged ≥19 years who visited any of the 17 primary care clinics between September 2020 and November 2020 and were diagnosed with hypertension, diabetes, dyslipidemia, or metabolic syndrome were included in this study. Patients who met the following criteria were excluded from the study: (1) having a condition that might compromise adherence to using mobile phones (such as those with visual or hearing limitations); (2) having comorbid conditions (such as breathing difficulties, uncontrolled congestive heart failure, or angina); (3) inability to communicate in the Korean language; and (4) those who were currently using or had used mobile self-management health care apps or weight loss medications within one month of the study. Physicians from 17 primary care clinics identified potential participants using medical records.

### Study Design

**Figure 1:**
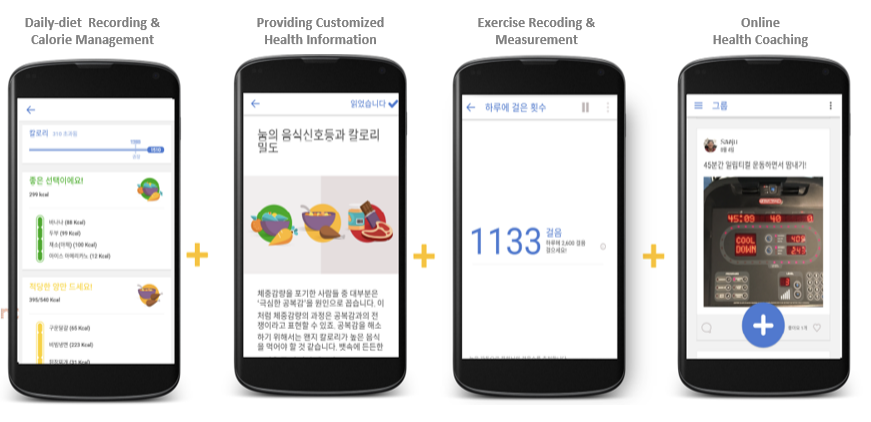
Example to track their daily activity, food intake, blood pressure, and blood sugar and chat human coaching.

This study is a multicenter real-world study that evaluated the effectiveness of the mobile self-management health care apps with combined primary care in the current primary care environment. The patients were divided into an app user group, which received a mobile self-management health care app called Noom (Noom Inc; Figure 1), and a control group. For group assignment, when a physician suggests to use the app to patient who needs continuous lifestyle modification during the treatment in the clinic, a researcher dispatched to the primary care center explains the intervention program to the patient and if the patient agrees to use the application assigned to the application use group, and if they did not agree, they were assigned to the control group. The goal of the Noom app is to enable users to lose weight and develop healthier habits via a behavioral approach. Users of the Noom app have access to built-in tools to track their daily activity, food intake, blood pressure, and blood sugar levels. It is one of the most used mobile self-management health care apps in South Korea and has been recognized by the Diabetes Prevention Program of the Centers for Disease Control and Prevention. The mobile self-management health care program used in this study lasted for 12 weeks and included human coaching sessions twice a week. The coaches were trained nutritionists who helped users set and implement achievable goals. Upon installation of the Noom app, the user answered a series of questions regarding their current weight, health problems, and lifestyle (such as, “Do you cook or eat out more?” and “How active you are during the day?”). The human coaches used patients’ responses to make dietary recommendations and to provide lifestyle advice to the patients.

The patients in the app user group recorded their diet and exercise using the app. They received personalized feedback and education from their human coach through mobile messages sent through the Noom app thrice a week, along with 1 or 2 primary care consultations over 12 weeks after the primary visit. For example, the human coach explains to the patients in the app user group what they are doing well and where they are not and sends related articles or videos. It also sets goals for the next step. The intervention program applied to the study is the same program as the existing Noom app program sold, but additionally, the participants shared their life log data recorded in the Noom app with their attending physician. The physicians received the app history data of participants in the form of reports from Noom on the launched website and provided professional feedback for lifestyle management in the clinic. The control group received conventional care, including lifestyle correction counseling to help self-manage chronic disease and providing a basic information booklet on chronic disease once or twice for 12 weeks.

### Study Outcome Measurement

The primary outcome of this study was the difference in weight loss between the 2 groups after 12 weeks. The secondary outcomes included differences in the changes in sleep condition, quality of life, depression, anxiety, stress, BMI, waist circumference, blood sugar levels, blood pressure, and blood lipid levels in the application user group after using the Noom app for 12 weeks.

Sleep conditions were evaluated by sleep quality and average sleep duration. The sleep quality was measured by patients using the following Likert Scale of five categories: (1) very bad, (2) bad, (3) neutral, (4) good, and (5) very good; furthermore, sleep duration was also recorded. The patients’ quality of life, indicating the extent to which patients are satisfied with their lives, was assessed using the Short Form-12 Health Survey (SF-12) questionnaire [27]. Depression was assessed using the Patient Health Questionnaire-2 (PHQ-2) [28], and anxiety was assessed using the Generalized Anxiety Disorder, 2-item (GAD-2) questionnaire [29]. Stress was assessed using the 10-level perceived stress scale [30]. Other measurements (body weight, BMI, and waist circumference) and laboratory tests (blood sugar levels, blood pressure, and blood lipid levels) were obtained at the participating primary care facilities and performed on only a subset of participating patients as judged necessary by the primary care physicians (64/110, 58.2%).

### Statistical Analyses

Baseline participant characteristics were compared between the study arms and tested for significance using the *t* test for continuous variables and chi-square test for categorical variables. An analysis of variance was used to identify differences between patients in the app user group who used the app for different lengths of time. Statistical significance was set at *P*<.05. Before analysis, propensity score (PS) matching was performed on variables such as age, sex, educational status, and underlying diseases to adjust the basic characteristics of the two groups. PS matching logit method was used at a ratio of 1:2. All statistical tests were 2-sided and conducted using Stata 16 (StataCorp).

### Ethical Considerations

The clinical research coordinator at Seoul National University Hospital explained the details of the study to the participants, and informed consent was obtained from each participant willing to participate. The institutional review board of Seoul National University Hospital approved the study (approval number H-2102-136-1199). The clinical study was conducted in accordance with the Good Clinical Practice guidelines and the tenets of the Declaration of Helsinki.

## Results

Although this study included 128 patients in the app user group and 50 patients in the control group who completed the program for 12 weeks (after recruiting a total of 218 patients from 17 primary care centers, those lost to follow-up were excluded), PS matching was conducted by considering the confounding as age, sex, educational status, and underlying diseases. The final analysis included 65 patients in the app user group and 45 in the control group after adjusting for baseline characteristics.

The baseline characteristics of the two groups were similar (Table 1). Although patients in the app user group were younger and had a higher level of education, the differences were not significant. Patient body weight (*P*<.001), sleep condition (sleep quality, *P*<.001; sleep duration, *P*<.001), stress (*P*=.01), BMI (*P*=.04), waist circumference (*P*=.03), HbA_1c_ levels (*P*=.04), high-density lipoprotein (HDL) cholesterol levels (*P*=.02), and triglyceride levels (*P*=.003) were significantly improved after 12 weeks in the app user group. There were no significant differences in the control group after 12 weeks (Tables 2 and 3).

**Table 1:** Demographic and clinical characteristics of participants (N=110).

Demographic description		Intervention group (n=65)	Control group (n=45)	*P* value	
Age (years), mean (SD)		51.93 (8.06)	55.24 (10.46)	.08	
**Sex, n (%)**					.39
	Male	25 (38.46)	21 (46.67)		
	Female	40 (61.54)	24 (53.33)		
**Education status, n (%)**					.07
	High school or lower	32 (49.23)	30 (66.67)		
	College or university	33 (50.77)	15 (33.33)		
**Disease or condition, n (%)**					.36
	Hypertension	22 (34.00)	15 (33.00)		
	Diabetes mellitus	35 (54.00)	24 (53.00)		
	Hyperlipidemia	3 (5.00)	3 (7.00)		
	Metabolic syndrome	5 (8.00)	6 (13.00)		

**Table 2:** Changes in clinical characteristics after 12 weeks.

Clinical characteristics		Intervention group (n=65), mean (SD)	*P* value		Control group (n=45), mean (SD)		*P* value	
**Body weight**				<.001				.63
	Baseline	78.10 (17.46)			74.05 (15.30)			
	After 12 weeks	76.67 (17.10)			73.92 (15.03)			
**Sleep quality**				<.001				.72
	Baseline	3.12 (1.00)			3.20 (0.89)			
	After 12 weeks	3.49 (0.95)			3.15 (0.87)			
**Sleep duration**				<.001				.65
	Baseline	6.23 (1.17)			6.18 (1.04)			
	After 12 weeks	6.59 1.12			6.14 (1.12)			
**Short Form-12 Health Survey (physical composite) score**				.12				.25
	Baseline	43.17 (4.51)			43.91 (4.63)			
	After 12 weeks	43.94 (5.01)			44.86 (4.82)			
**Short Form-12 Health Survey (mental composite) score**				.12				.03
	Baseline	39.91 (5.56)			38.46 (5.98)			
	After 12 weeks	41.04 (4.97)			40.83 (7.60)			
**Patient Health Questionnaire-2 score**				.87				.86
	Baseline	1.29 (1.71)			0.88 (1.54)			
	After 12 weeks	1.26 (1.53)			0.93 (1.48)			
**Generalized Anxiety Disorder-2 scale score**				.26				.49
	Baseline	1.16 (1.68)			0.80 (1.37)			
	After 12 weeks	0.98 (1.52)			0.66 (1.39)			
**Propensity score**				.01				.92
	Baseline	16.38 (6.89)			14.02 (5.61)			
	After 12 weeks	14.43 (5.9)			14.11 (6.03)			

**Table 3:** Changes in physical and laboratory measurements after 12 weeks^a^.

Measurements		Intervention group (n=42), mean (SD)	*P* value		Control group (n=24), mean (SD)		*P* value	
**Waist circumference** ** **				.03				.53
	Baseline	93.41 (11.09)			89.99 (9.08)			
	After 12 weeks	91.75 (11.57)			89.64 (9.14)			
**BMI**				.04				.66
	Baseline	28.60 (4.47)			27.65 (4.12)			
	After 12 weeks	27.83 (4.23)			27.58 (4.04)			
**Systolic blood pressure**				.09				.76
	Baseline	123.95 (12.35)			127.75 (12.02)			
	After 12 weeks	127.02 (15.00)			126.75 (10.77)			
**Diastolic blood pressure**				.06				.76
	Baseline	77.71 (7.83)			77.87 (8.30)			
	After 12 weeks	81.19 (11.51)			78.37 (9.70)			
**Hemoglobin A_1c_ levels**				.04				.89
	Baseline	6.69 (1.06)			6.73 (0.94)			
	After 12 weeks	6.51 (0.91)			6.72 (0.93)			
**Total cholesterol**				.83				.73
	Baseline	165.80 (46.49)			159.29 (36.22)			
	After 12 weeks	167.26 (35.68)			157.08 (41.57)			
**High-density lipoprotein cholesterol levels**				.02				.67
	Baseline	48.50 (11.30)			47.17 (10.82)			
	After 12 weeks	51.23 (12.36)			47.78 (8.04)			
**Low-density lipoprotein cholesterol levels**				.37				.54
	Baseline	85.83 (30.32)			78.73 (30.13)			
	After 12 weeks	89.49 (30.97)			76.01 (35.83)			
**Triglyceride levels**				.003				.47
	Baseline	176.38 (109.02)			193.33 (199.90)			
	After 12 weeks	136.07 (54.2)			166.37 (60.93)			

^a^All physical and laboratory measurements were obtained at the participating primary care center and only for a subset of participating patients as judged necessary by the primary care physicians. Hence, this table shows only 42 and 24 participants in the intervention and control groups, respectively.

### Primary Outcome

The app user group had significantly more weight loss than the control group: the body weight of the app user group decreased by 1.43 kg (95% CI –2.07 to –0.79) and that of the control group decreased by 0.13 kg (95% CI –0.67 to 0.41; *P*=.002; Table 4). Patients in the app user group who used the Noom app for at least 9 weeks had a significantly higher weight loss than those who used the app for 5-8 weeks and those who used the app for ≤4 weeks (*P*=.002; Table 5).

**Table 4:** Comparison of measurements changes between baseline and after 12 weeks by the group.

Measurements	Intervention group (n=65), mean (SD)	Control group (n=45), mean (SD)	*P* value
Body weight (kg)	–1.43 (2.59)	–0.13 (1.78)	.002
Sleep quality score	0.36 (0.71)	–0.04 (0.82)	.007
Sleep duration (hours)	0.35 (0.78)	–0.04 (0.64)	.004
Short-Form-12 Health Survey (physical composite) score	1.91 (5.64)	0.94 (5.44)	.37
Short-Form-12 Health Survey (mental composite) score	1.12 (5.74)	2.37 (7.21)	.34
Patient Health Questionnaire-2 score	–0.03 (1.46)	0.04 (1.71)	.81
Generalized Anxiety Disorder-2 scale score	–0.18 (1.30)	–0.13 (1.27)	.84
Propensity score	–1.95 (6.01)	0.08 (5.99)	.08
Waist circumference (cm)^a^	–1.82 (4.53)	–0.15 (3.59)	.04
BMI (kg/m^2^)^a^	–0.53 (0.99)	–0.04 (0.64)	.002
Systolic blood pressure (mm Hg)^a^	3.35 (11.02)	1.23 (17.36)	.51
Diastolic blood pressure (mm Hg)^a^	3.23 (11.34)	0.05 (8.78)	.14
Hemoglobin A_1c_ levels (%)^a^	–0.17 (0.60)	–0.01 (0.42)	.14
Total cholesterol levels (mg/dl)^a^	4.09 (42.65)	–0.82 (27.43)	.52
High-density lipoprotein cholesterol levels (mg/dL)^a^	3.16 (7.10)	2.03 (6.99)	.47
Low-density lipoprotein cholesterol levels (mg/dL)^a^	6.99 (30.85)	–0.94 (20.28)	.16
Triglyceride levels (mg/dL)^a^	–37.94 (89.18)	–51.05 (182.82)	.70

^a^All physical and laboratory measurements were obtained at the participating primary center and only for a subset of participating patients (intervention group, n=42; control group, n=24) as judged necessary by the primary care physicians.

**Table 5:** Comparison of measurements change between baseline and after 3 months by app use period.

Measurements	Control group (n=45), mean (SD)	Intervention group (n=65), mean (SD)				*P* value
		Less than 4 weeks (n=18)	5-8 weeks (n=11)	Greater than 9 weeks (n=36)		
Body weight (kg)	–0.13 (1.78)	–0.90 (1.75)	–0.33 (1.55)	–2.02 (3.03)	.002	
Sleep quality score	–0.04 (0.82)	0.16 (0.38)	0.27 (0.46)	0.50 (0.87)	.02	
Sleep duration (hours)	–0.04 (0.64)	0.25 (0.55)	0.36 (0.77)	0.40 (0.89)	.045	
Waist circumference (cm)	–0.15 (3.59)	–1.18 (1.93)	–1.37 (1.93)	–2.21 (5.84)	.22	
BMI (kg/m^2^)	–0.04 (0.64)	–0.33 (0.69)	–0.13 (0.64)	–0.75 (1.15)	.003	

### Secondary Outcomes

The subjective assessments of sleep quality (*P*=.007), sleep duration (*P*=.004), waist circumference (*P*=.04), and BMI (*P*=.002) were significantly more favorable in the app user group than in the control group after 12 weeks (Table 4). Patients in the app user group tended to display a greater change in stress scores than those in the control group, although the differences were not significant after 12 weeks (Table 4). The sleep conditions and BMI also improved as patients in the app user group using the app for 9 weeks or more (Table 5).

## Discussion

### Principal Findings

After 12 weeks of using the Noom app, patients in the app user group reported significantly greater weight loss and improved sleep quality and duration than those in the control group. This is the first Korean study to determine the efficacy of a mobile app for the self-management of chronic conditions in the current primary care setting.

The results of this study are similar to those of a previous study regarding the use of an app and medical provider management for patients with obesity and hypertension, diabetes, or hyperlipidemia [31]. The weight loss observed in this study was greater than that reported previously when considering the duration of both these studies. This is likely since this study combined the use of a mobile app with human coaching to enhance the patients’ self-management competency. The Opportunities for Weight Reduction (POWER) study combined telephone-based coaching and web-based training modules with self-managed interventions and resulted in weight reduction that was comparable to that in this study [32]. Taken together, the results of these studies suggest that a self-management program using a mobile app, human coaching, and provider counseling is effective for weight loss in patients with hypertension, diabetes, or hyperlipidemia. Furthermore, the results of this study show that the results of these studies are also linked to the current primary care setting.

Several previous studies have evaluated the effectiveness of mobile self-management health care apps with varied results. While some studies have shown that using mobile self-management health care apps is effective for weight loss in patients with chronic diseases and improving blood pressure control, total cholesterol and triglyceride levels, and waist circumference [33-36]. However, results from another study indicate that the weight change was minimal and insignificant compared with a control group [37]. In addition, the use of mobile self-management health care apps allowed patients to maintain a healthy lifestyle after 12 months, though long-term differences between the app user groups and the control groups were not reported in a previous study [38]. Previous South Korean studies on the effectiveness of mobile self-management health care apps focused on weight loss [39,40].

The Noom app can be used to alleviate the difficulties related to continuous self-monitoring, provide patient education, customize feedback [41], and manage meetings with physicians. However, to make the intervention cost-effective, a low-cost digital technique (interactive text, questions and answers, and feedback text messaging) was used in this study. This method may limit the use in the elderly population that is on the rise. In addition, even when the low-cost digital methods of communication were used, the maintenance cost of the methods used in this study was higher than the cost of using only the app. This resulted from the providing app and provider counseling that involved a human coach. Previous studies have shown that the inclusion of human counseling results in more favorable outcomes when using digital health interventions [42,43]. Patient data algorithms were used in this study to analyze patient data to produce short medical consultation reports for clinicians, data dashboards that organize the coaching process and show the patients’ lifestyles, and personalized feedback for the patients.

### Limitations and Strengths

This study has several strengths. The greatest advantage of this study was that it involved a multicenter primary clinic of the current primary care setting. This has shown that the primary care services combining a mobile self-management health care app with human coaching are effective in the current primary care system where there are barriers such as patient perception. Second, it was based on using a mobile phone app instead of a web-based program. Mobile phone interventions result in increased involvement of the participant compared with web-based programs. Third, the human coaching component included in this study provided individualized, one-on-one feedback based on self-monitoring data provided by the patient. When the patient entered data on his/her diet and activities, the coach confirmed the data and helped the patients use the app better and improve their health management skills via individualized feedback. Last, the human coaches provided summary data to the primary care provider, allowing the primary care staff to provide feedback regarding the patient’s lifestyle while providing medical care. This allowed for the reinforcement of lifestyle modifications from various sources and extended the responsibilities of the medical staff.

However, this study also had some limitations. The first limitation of this study is the nonrandomized. This could lead to a selection bias based on mobile approach properties, and patients who participated in the application user group actually tended to be younger and more educated than the control group. To compensate for this limitation, this study attempted to minimize this bias by implementing PS matching by adjusting the basic characteristics such as age, sex, educational status, and underlying diseases. Nevertheless, it may have influenced the assignment of patients to interventions and controls. Second, owing to the study design, it was not possible to isolate the specific effects of each component of the intervention. It may be unclear whether the effect of the study is a representation on the use of mobile self-management health care app, an effect on human coaching, or an effect on primary care. Finally, the sample size is small for generalization.

### Conclusions

The combination of primary care services combining a mobile self-management health care app with human coaching is more effective than conventional primary care for weight loss and improving sleep in patients with chronic diseases in primary care clinics. An implication of this is the possibility that a mobile self-management health care app with human coaching is a treatment option in the current primary care system. In the future, for a mobile self-management health care app to become a general treatment option, large-scale randomized studies on the long-term effects of interventions in the current primary care settings are needed.

## Abbreviations

GAD-2: Generalized Anxiety Disorder, 2-item
HbA_1c_: hemoglobin A_1c_
HDL: high-density lipoprotein
PHQ-2: Patient Health Questionnaire-2
POWER: Opportunities for Weight Reduction
PS: propensity score
SF-12: Short Form-12 Health Survey
